# Extensive Reliability Evaluation of Docking-Based Target-Fishing Strategies

**DOI:** 10.3390/ijms20051023

**Published:** 2019-02-27

**Authors:** Margherita Lapillo, Tiziano Tuccinardi, Adriano Martinelli, Marco Macchia, Antonio Giordano, Giulio Poli

**Affiliations:** 1 Department of Pharmacy, University of Pisa, 56126 Pisa, Italy; tiziano.tuccinardi@unipi.it (T.T.); adriano.martinelli@unipi.it (A.M.); marco.macchia@unipi.it (M.M.); 2 Sbarro Institute for Cancer Research and Molecular Medicine, Center for Biotechnology, College of Science and Technology, Temple University, Philadelphia, PA 19122, USA; giordano@temple.edu; 3 Department of Medical Biotechnologies, University of Siena, 53100 Siena, Italy

**Keywords:** target fishing, docking, reverse screening, consensus docking

## Abstract

The development of target-fishing approaches, aimed at identifying the possible protein targets of a small molecule, represents a hot topic in medicinal chemistry. A successful target-fishing approach would allow for the elucidation of the mechanism of action of all therapeutically interesting compounds for which the actual target is still unknown. Moreover, target-fishing would be essential for preventing adverse effects of drug candidates, by predicting their potential off-targets, and it would speed up drug repurposing campaigns. However, due to the huge number of possible protein targets that a small-molecule might interact with, experimental target-fishing approaches are out of reach. In silico target-fishing represents a valuable alternative, and examples of receptor-based approaches, exploiting the large number of crystallographic protein structures determined to date, have been reported in the literature. To the best of our knowledge, no proper evaluation of such approaches is, however, reported yet. In the present work, we extensively assessed the reliability of docking-based target-fishing strategies. For this purpose, a set of X-ray structures belonging to different targets was selected, and a dataset of compounds, including 10 experimentally active ligands for each target, was created. A target-fishing benchmark database was then obtained, and used to assess the performance of 13 different docking procedures, in identifying the correct target of the dataset ligands. Moreover, a consensus docking-based target-fishing strategy was developed and evaluated. The analysis highlighted that specific features of the target proteins could affect the reliability of the protocol, which however, proved to represent a valuable tool in the proper applicability domain. Our study represents the first extensive performance assessment of docking-based target-fishing approaches, paving the way for the development of novel efficient receptor-based target fishing strategies.

## 1. Introduction

The development of target-fishing (TF) approaches, aimed at identifying the possible protein targets of a small molecule, still represents a current topic in medicinal chemistry. Computational approaches are conventionally focused on studying the interactions of multiple drug-like molecules with a single protein target, and they are successfully employed in virtual screening (VS) campaigns for identifying novel ligands for the target of interest [1,2]. Differently, computer-aided reverse screening methods, also known as in silico TF, are increasingly being used to identify the most likely protein target of a query ligand [3]. TF methods are highly valuable for predicting the bioactivity of a query small molecule, or elucidating the mechanisms of action of all therapeutically interesting compounds, for which the actual target is still unknown. Therefore, TF strategies have found multiple applications in the fields of drug discovery and biomedical research [4]. 

Reverse screening approaches also represent important computational techniques for identifying new macromolecular targets of existing drugs or active compounds, and for analyzing their functional mechanisms or side effects [5]. In fact, in silico TF strategies can find application in drug repositioning campaigns, thus saving huge amount of money that have been estimated for the successful launch of a single new drug [6,7,8], as well as in off-target effect predictions [9,10]. However, off-targets can also be responsible for the beneficial secondary effects of existing drugs and drug candidates. It has been proven that each known drug has, on average, six different molecular targets on which it exhibits activity [11]. In this sense, polypharmacology, i.e., the ability of for small molecules to interact with multiple protein targets, acquire particular interest for rationally designing more effective and less toxic drugs [12]. Actually, polypharmacology can be highly desirable in the treatment of cancer and other complex diseases that involve the functional modulation of multiple proteins [13].

Due to the huge number of possible protein targets that a small molecule may interact with, experimental TF approaches are out of reach, since they involve time-consuming, and above all, expensive biological assays [14]. Taking into consideration the continuous development of computational techniques, in silico TF strategies represent a valuable alternative to classic high-throughput screening (HTS) approaches. These computational methods may be divided into two classes according to their underlying principles: ligand-based methods such as shape-based screening and pharmacophore screening, and receptor-based strategies, namely reverse docking [15]. In the absence of receptor X-ray structures, the above-mentioned ligand-based methods allow for the identification of potential protein targets of a query molecule, based on the hypothesis that similar ligands bind similar targets. Therefore, either the molecular structure or the shape of the query molecule, or its key pharmacophore features, are compared with those of compounds that are known to be active toward certain targets [16]. Then, the known targets of the ligands that best satisfy the similarity criteria can be considered as potential targets of the query molecule. The advantage of ligand-based TF approaches relies on the fact that no structural knowledge of the target receptors is needed for their application. However, only protein targets for which active compounds have been experimentally identified and reported in literature can be taken into account, using these approaches. Moreover, the efficacy of these methods is hampered by the structural diversity between the query molecule and the known ligands; therefore, a true target of the query ligand is likely to be successfully predicted only if structurally related active compounds have already been discovered.

Conversely, receptor-based methods only rely on the structural information that are relative to the potential target receptors. In fact, reverse docking consists of evaluating the possible binding mode of the query molecule into the binding site of multiple protein targets, in order to identify proteins with strong binding affinities for the query ligand that can thus be considered as its potential targets [17]. Therefore, when exploiting the large number of crystallographic protein structures that have been determined to date [18], such a receptor-based approach represents an effective strategy for the target prediction of a query ligand. Reverse docking approaches indeed require only the availability of a single structure for each target to be screened, and they can be applied, regardless of the presence/absence of the known ligands for the test targets. Moreover, reverse docking appears to be the most comprehensive method, since it considers the key elements of both molecular shape and the pharmacophore moieties of the query ligand in relation to the binding sites of the screened targets. Several examples of receptor-based TF approaches have been reported in the literature [19,20]. However, to the best of our knowledge, no proper evaluation of such an approach has been performed yet. 

In the present study, taking into consideration the high potential of a reverse docking strategy in identifying the most likely target of a query ligand, an extensive performance assessment of docking-based TF approaches was carried out. For this purpose, a set of X-ray structures belonging to different targets was selected, and a dataset of compounds, including 10 experimentally active ligands for each target, was created. A target-fishing benchmark database was thus obtained and used to assess the reliability of 13 different docking procedures in identifying the correct target of the dataset ligands.

## 2. Results and Discussion

To assess the reliability of a docking-based TF strategy, we created a benchmark database, including the X-ray structure of 60 different targets and 600 known active compounds. The selected targets and their ligands belonged to three datasets that have been broadly used in the validation of computer-aided drug design methods, i.e., the Directory of Useful Decoys (DUD) [21], the Maximum Unbiased Validation (MUV) [22], and ChEMBL datasets [23] (see Materials and Methods for details). The 60 selected targets covered a wide range of protein types, since they comprised steroid hormone receptors (androgen, estrogen, glucocorticoid, mineralocorticoid and progesterone receptors), different enzymes, including many kinases and hydrolases, some reductases or phosphorylases, several transmembrane receptors coupled to G proteins (adrenergic, dopaminergic or muscarinic receptors), and other different protein targets (Table 1). For each target, 10 active ligands were chosen among the experimentally active compounds reported in the corresponding datasets, considering some structural variability among them (where possible), in order to avoid any bias in docking results due to the potential structural similarities of the ligands.

**Figure 1:**
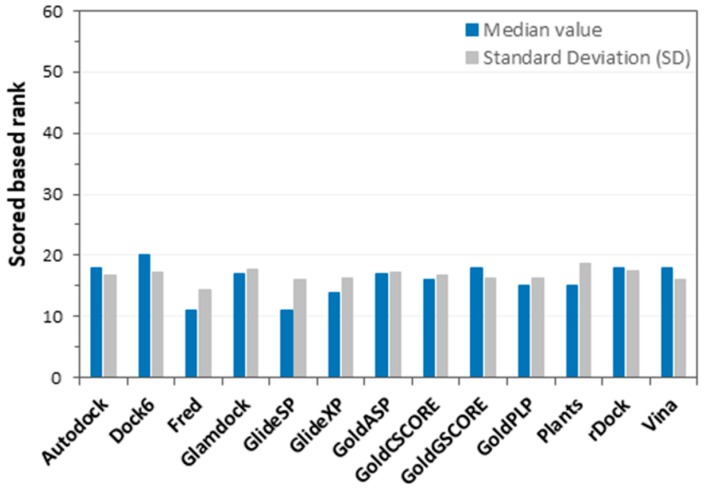
Results of the first docking score analysis. The blue bars represent the median ranking positions of true targets, calculated for each docking procedure by computing the median value of the single score ranking positions obtained for each ligand (600 ligands docked into 60 different targets). Grey bars stand for SD; namely, the dispersion of the ranking position values calculated for the 600 ligands in each docking procedure.

As a first step, we evaluated the ability of every single docking procedure to identify the proper target of each dataset ligand. Therefore, the 600 compounds were docked into the X-ray structures of all of the selected targets, and for each ligand, the docking result obtained in its “correct” target was compared with those generated by the docking calculations in the other targets. This protocol was applied by using 13 different docking procedures (see Materials and Methods for more details), and as a result, a total of about 470,000 docking calculations were taken into account. The docking score value relative to the best-ranked docking pose calculated for each ligand was considered as a parameter to compare docking results. Basically, the docking score is a measure of the ligand–protein binding affinity that is estimated by the docking methods, taking into account the number and type of favorable intermolecular interactions established by the molecule within the protein binding site in the predicted docking pose [24,25]. For each ligand, the 60 docking score values associated with the docking poses obtained with the 60 targets were employed to rank the potential affinities of the 60 targets for the ligand; then, the ranking position of the true target of the ligand was calculated and used for statistical evaluations. In fact, in the ideal case, the true target of the ligand should present the maximum affinity (and thus the highest rank), since the score associated with the docking pose of the ligand into its true target should be higher than those that are associated with the docking poses of the same ligand into different targets. To assess the performance of every single docking method in identifying the correct target of each ligand, and to compare the results obtained from different docking procedures, we calculated the median ranking position of the ligands’ own targets that were achieved by using each docking method (see Materials and Methods for more details). Figure 1 summarizes the main results obtained from this first docking analysis. Fred and Glide, using the standard precision (SP) method, seemed to be the best performing docking procedures, as they both showed a median ranking position of the true targets of 11.0, out of 60 total targets. This means that, considering the target fishing screens performed by using each of the 600 dataset ligands as the query molecule, the correct target of the query ligands was ranked 11th overall in the targets dataset. On the contrary, Dock6 showed the worst performance, with a median ranking position of 20.0. Despite these differences, the results obtained did not allow for the identification of a single promising docking procedure that was able to recognize the correct target of a ligand in an effective manner. In fact, the calculated median values revealed that the different docking procedures ranked the correct target of each ligand at around the top 20–30% of the target dataset. Moreover, it is worth noting that a high standard deviation (SD), namely a large variability of ranking position values, was observed for every tested docking procedure, indicating that the obtained results were spread out over a wide range of values (Figure 1). This may be ascribed to the intrinsic variability of the docking results in terms of docking poses and scores that are produced by single methods for different ligands and targets, as already observed in our previous validation analyses of docking procedures across different targets [26,27,28].

The target-fishing performances of the different docking procedures were also evaluated in terms of the true positive rate (TPR) and false discovery rate (FDR), in order to better verify the quality of target prediction achieved by using the different docking methods (see Materials and Methods for details). Specifically, the TPR is a measure of the overall target prediction reliability. In fact, the higher the TPR obtained by using a certain docking procedure, the higher the number of predictions in which the correct target of the query ligand was ranked within the top 10% of the target dataset. Conversely, the FDR is a measure of target prediction inaccuracy, since the higher its value, the higher the overall number of incorrect targets ranked within the top 10% of the dataset. As shown in Table 2, the percentage of TPR achieved by the tested procedures ranged from a minimum value of 22%, obtained using Vina, up to a maximum value of 36% achieved by Fred and Glide SP, which were confirmed to be the best performing procedures. This means that, by using these two docking methods, true target predictions were obtained for 36% of the query ligands (i.e., for 213 out of the 600 dataset ligands). On the other hand, high FDR values were found for all the different docking procedures, ranging between 67% and 81%. Again, Fred and Glide SP showed the best results, being the only two procedures with an FDR below 70%; however, these values still highlight a certain overall inaccuracy of the target prediction, which is consistent with the high standard deviation that is observed in the results of the different docking methods. Overall, this analysis confirmed the results highlighted by the first evaluation, based on target ranking.

**Figure 2:**
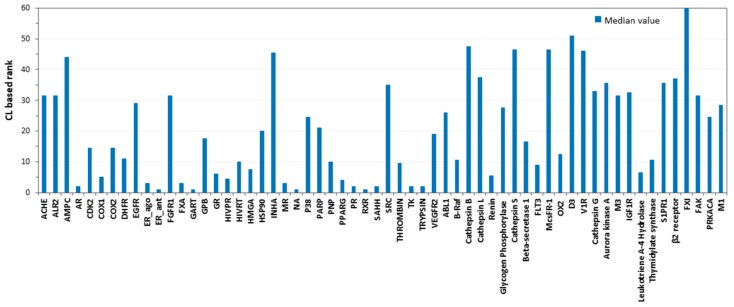
Results of the consensus docking (score) analysis. Blue bars represent the ranking position (based on the consensus level, CL) estimated for the proper target of each ligand, with respect to the others. In this picture, the median values of the target ranking positions obtained for the 10 ligands belonging to the same target were calculated.

To evaluate whether combining the results of multiple docking procedures could lead to an improvement in target prediction capability, a consensus docking analysis [29] was performed. As shown by previous results, a consensus docking approach can be profitably used to predict reliable ligand binding dispositions [30], and to identify new hit compounds in virtual screening strategies [31]. In this instance, we were interested in the effects of a consensus docking analysis on the ability to identify the correct targets of a ligand. The docking score was again used as the evaluation parameter; thus, a consensus scoring approach was basically followed in these analysis. In particular, we calculated the number of docking methods (among the 13 methods used) that were able to rank the proper targets of each ligand to within the top-scored 10% of the total targets, defined as the consensus level (see Materials and Methods for more details). The same analysis was applied to the 59 unrelated targets of each ligand, and for all of them, the consensus level was also calculated. The ranking position of the proper target of each ligand with respect to the other targets was then estimated, based on the consensus level obtained. As shown in Table 2, the consensus docking analysis confirmed the previously obtained results. Basically, the combination of the results obtained by the 13 docking procedures did not cause an actual improvement in target prediction ability, although it performed as well as the best methods tested, achieving a TPR of 36% and an FDR of 67%. Moreover, it was observed that there was a considerable variability among the results achieved for the different targets. For instance, AR, SAAH, and TK were identified as being the most likely targets of their corresponding active ligands, being ranked within the first two positions of the targets dataset (Figure 2). Conversely, INHA, D3, and FXI were only ranked among the last 15 positions of the targets dataset (rank 45, 51 and 60 respectively); therefore, they were not identified as being possible targets of the corresponding ligands.

Consistent with these results, we observed a clear difference among the consensus level values that was achieved by the different targets (Table 3). We envisioned that this diversity in docking performances might rely in the different types of ligands and proteins herein taken into account. Based on these considerations, we investigated whether some properties of the targets and/or ligands could affect docking results, thus influencing the ability of the applied docking procedures (either alone or in combination) in identifying the true target of a ligand.

**Figure 3:**
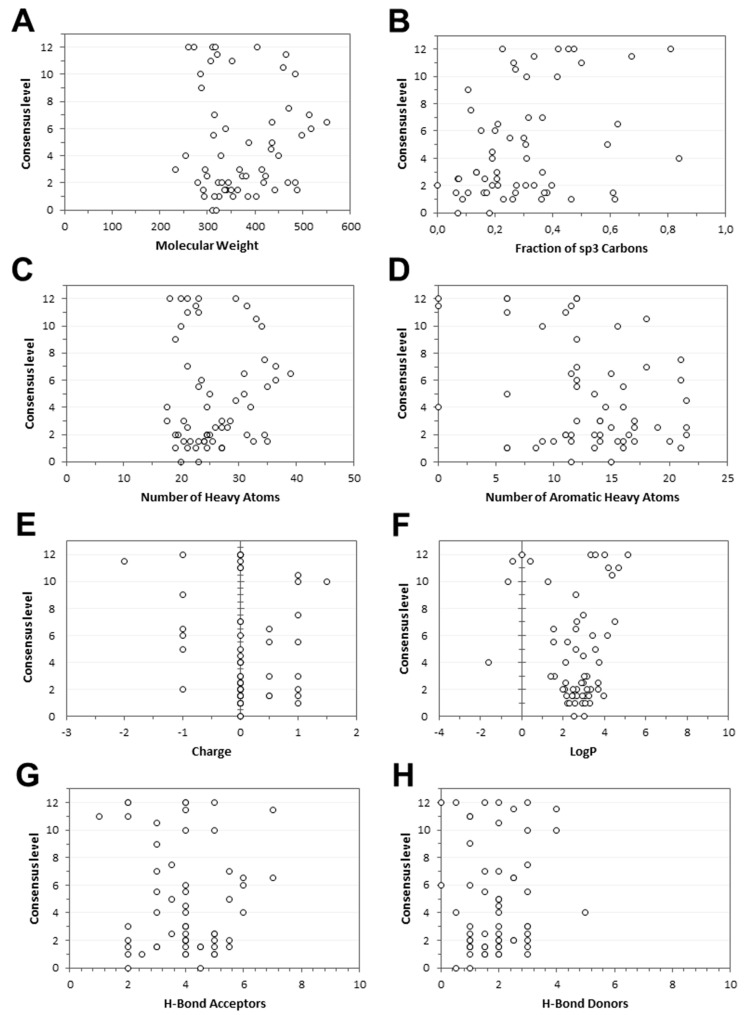
Analysis of the consensus docking results in relation to the ligand properties. (**A**) The molecular weight, (**B**) fraction of sp3 carbons, (**C**) number of heavy atoms, (**D**) number of aromatic heavy atoms, (**E**) charge, (**F**) log*P*, (**G**) number of H-bond acceptors, and (**H**) number of H-bond donors are reported in the graphs, respectively.

Regarding ligands, both the molecular weight (MW) and the number of heavy atoms were considered, in order to evaluate whether the sizes of the different molecules could affect docking results. Moreover, the effects of charged moieties, hydrogen bonds acceptors, and hydrogen bond donors in the dataset ligands were evaluated. To verify whether the consensus level could be positively or negatively affected by the conformational freedom of a molecule, we calculated the number of aromatic heavy atoms, and the fraction of sp3 carbons in all the tested compounds. Finally, we evaluated the effects of the ligand lipophilicity on the consensus level. For this purpose, the consensus log*P* value of the dataset ligands, which combines five different log*P* calculation methods, was obtained through the Swiss ADME web tool [32], as previously performed [33]. The median value of each property, calculated for the 10 ligands belonging to each target, was related to the median consensus level that was achieved by the same target. As shown in Figure 3, no evident link was observed between the eight considered ligand properties and the consensus level that was reached by targets. Concerning the net charge of the ligands (Figure 3E), it is worth noting that a high consensus level (from 10 to 12) frequently corresponded to clusters of ligands characterized by a common charged group (all negative or positive), suggesting that such a group potentially represents an essential feature for the ligand–protein interaction, and it has an effect on ligand binding affinity. However, no linear trend that was able to justify a clear relationship between the charge and the consensus level was observed (see also Figures S1–S4 in the Supplementary Materials). 

**Figure 4:**
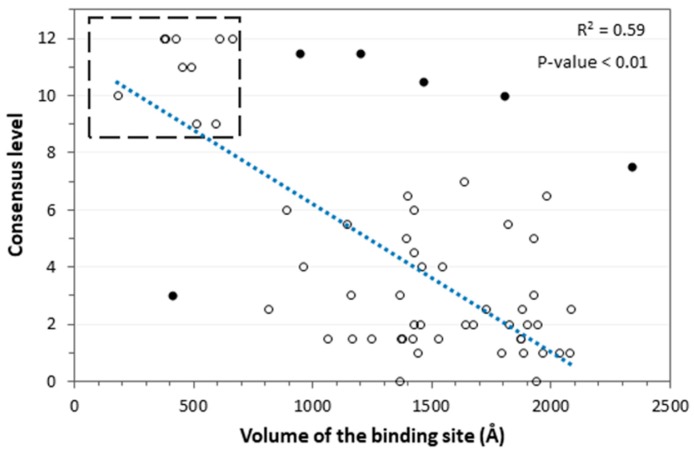
Analysis of the consensus docking results in relation to the target properties. The trend line is displayed as a dashed blue line. Closed black circles represent targets that did not show any link between the volume of the protein binding site and the consensus level.

Regarding the targets, the volumes of the binding sites were taken into consideration, with the aim of evaluating whether a different size of the target binding pockets could affect the docking results. In this case, an interesting trend was observed, since the consensus level tends to be higher for targets with small and mainly closed binding pockets. Figure 4 shows the results obtained from this analysis. In particular, as the volume of the binding sites increased, and the binding pockets became more open and solvent-accessible, the consensus level decreased, emphasizing the tight connection between these properties and the target prediction ability of the docking procedures. The 10 targets that showed a higher consensus level (open circles enclosed within the dashed square in Figure 4) belonged to the class of steroid hormone receptors (androgen, estrogen, glucocorticoid, mineralocorticoid and progesterone receptors) and other classes of proteins (COX1, HIVRT, RXR, SAHH, TK) that all shared small and mainly closed binding sites. Conversely, few targets (closed dark circles in Figure 4) significantly diverged from the common linear trend, namely NA, ER_ANT, FXA, GART, trypsin, and PNP. For these proteins, the reported consensus level was not found to be related to the target properties. However, we observed that the reference active ligands of all of these targets shared a common structural moiety. For instance, the NA and GART ligands presented a negatively charged moiety, while the ER_ANT, FXA and trypsin ligands were characterized by a positively charged group. As shown in Figure 4, a high consensus level (8 or above) was achieved by all of these targets; we thus hypothesized that these results were most probably due to the presence of the common charged portion that was shared by all active ligands of the same target, which probably affected the docking results (see also Figure 3E). Differently, the PNP ligands did not share a charged moiety; nevertheless, they all presented a common structural portion that might have influenced the docking results as well, although in a negative way. By excluding these six presumed outliers, a correlation coefficient of 0.59 between binding site volume and consensus level was obtained, with a *P*-value < 0.01.

**Figure 5:**
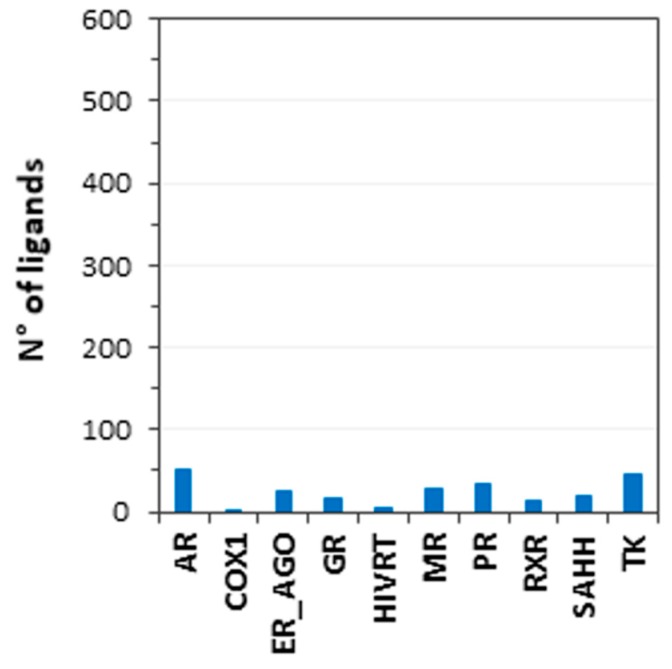
Number of dataset ligands for which target proteins with small binding sites achieved consensus levels of greater than or equal to 10.

Based on these results, the consensus docking-based TF procedure seemed to be effective for identifying the true targets of a ligand, when its corresponding receptor was characterized by a small and mainly closed binding site. In order to verify the reliability of these results, we calculated the number of ligands among the 600 dataset compounds, for which the targets with small binding sites achieved a high consensus level (above or equal to 10). In this way, we wanted to check whether the results of the consensus docking-based TF procedure were affected by a bias, due to the fact that high consensus levels were always achieved by targets with closed binding sites, regardless of the fact that the query molecule was a true active ligand of that target, or a decoy. Nevertheless, we observed that the targets with small binding sites obtained a high consensus level only for a maximum of 50 out of the 600 ligands, corresponding to less than 10% of the cases (Figure 5). Moreover, we verified that no single protein target reached a median consensus level (calculated by computing the median value obtained for the whole dataset of ligands) higher than 4. These analyses confirmed the reliability of the consensus docking-based TF protocol, at least for predictions involving targets characterized by a small or closed binding site. In practice, our evaluations demonstrated that if the consensus docking-based TF protocol is applied for identifying the possible targets of a certain query molecule, and a receptor characterized by a small or closed binding site is obtained among the top-scored targets, such a prediction should be considered as reliable, and the query molecule is likely to be an actual ligand of the identified target. On the contrary, the prediction of a protein presenting a large or highly solvent-exposed binding site as a possible target of the query molecule should be taken with caution, since it is probably not sufficiently reliable.

## 3. Materials and Methods

### 3.1. Database Generation

To evaluate the use of different docking methods in a target fishing (TF) strategy, we analyzed the broadly used Directory of Useful Decoys (DUD) [21], the Maximum Unbiased Validation (MUV) [22] and the ChEMBL dataset [23]. All targets presenting metal ion prosthetic groups were not considered in this study, since the presence of ions within the protein binding site could negatively affect the performance of some of the docking methods applied in the TF validation protocol. Similarly, all targets for which an X-ray crystal structure was not available (unresolved protein or homology model) were excluded from the analysis. Among the available X-ray crystal structures, only those that were co-crystalized with a ligand bound into the protein binding site were considered. Moreover, targets common to more than one dataset (DUD, MUV and ChEMBL) were taken into consideration only once. Therefore, a total of 60 different targets were selected; in particular, 35 targets belonged to DUD, six were included in MUV, and the last 19 were part of the ChEMBL dataset. For each target considered in this study, 10 experimentally active compounds were randomly selected among the active ligands reported in the corresponding database; conversely, decoy compounds were not considered in this study. A benchmark database of 600 ligands was thus created and used to assess the performance of the 13 different docking procedures described below. Such database could be considered as an enriched dataset, since it includes, for each target, 10 active compounds and a total of 590 different potential decoys.

### 3.2. Protein Structure Alignment

A local structure alignment and a superimposition of the binding site of the 60 targets used in the study has been carried out using Chimera [34]. All the residues outside a range of 10 Å from the X-ray ligand were removed; then, the different binding sites were aligned by using the Needleman–Wunsch alignment algorithm of the MatchMaker tool included in Chimera. No high homologies among the binding sites of the 60 targets was been found, except for three pairs of proteins, namely the two cyclooxygenase receptors COX 1 and COX 2, the two muscarinic receptors M1 and M2, as well as the two estrogen receptor alpha structures with PDB codes 1L2I and 3ERT, which have been both considered for the analysis, as they differ for the classes of co-crystallized and active ligands (respectively, agonists and antagonists). 

### 3.3. Docking Procedures

For all docking calculations, only the best scored pose was taken into account in the analysis. 

Autodock 4.2.3: AUTODOCK Tools utilities [35] were employed, in order to identify the torsion angles in the ligands, to add the solvent model, and to assign the Gasteiger atom charges to the protein and ligands. The region of interest used by the software was defined by considering the reference ligand as being the center of a grid box of 10 Å in the x, y, and z directions. A grid spacing of 0.375 Å, and a distance-dependent function of the dielectric constant were employed for the energetic map calculation. By employing the Lamarckian genetic algorithm, the 600 selected compounds were subjected to 20 runs of the AUTODOCK, using 2,500,000 steps of energy evaluation, and the default value of the other parameters.

Dock 6.7: The molecular surface of the binding site was calculated by the means of MS, creating a Connolly surface with a probe of 1.4 Å radius. By means of the Sphgen program, the points of the surface and the vectors normal to it were used to build a set of spheres with radii varying from 1.4 to 4 Å, which describe the negative image of the site from a stereoelectronic point of view. Spheres within a radius of 10 Å from the reference ligand were employed to identify the docking site. For each ligand, the software calculated 1000 orientations; among these, the best grid scored among them was considered in this study [36]. Ligands charges were calculated by employing the AM1-BCC method, implemented in the MOLCHARGE program.

Fred 3.0. A set of conformers, required by the software for each input ligand, was generated by OMEGA2. Standard values were used for all sampling parameters, except for the energy window (50.0), the maximum number of output conformers (10,000), the time limit (1200), and the RMSD value, below which two conformations were considered to be similar (0.3 Å). The region of interest for the docking calculations was determined, so that it contained all of residues located within 10 Å from the ligand in the X-ray structure. The FRED [37] docking calculation consists of a preliminary shape-fitting step, during which the ligand is placed into the binding site, using a smooth Gaussian potential and a subsequent optimization phase including (a) the refinement of the positions of the ligand’s hydroxyl hydrogen atoms (b) rigid body optimization (c) optimization of the ligand pose in the dihedral angle space. In the last optimization step, the Chemgauss3 scoring function was employed, and after the docking calculation, the poses were scored independently by Chemgauss4. FRED default parameters were used, imposing high dock_resolution.

Glamdock 1.0. The GLAMDOCK docking protocol consisted of five docking runs, each comprising 650 Monte Carlo minimization (MCM) steps, with 15 steps of Levenberg–Marquardt minimization in torsion space at each MCM step. Finally, a maximum of 40 poses were finally post-minimized by 150 steps of Levenberg–Marquardt [38].

Glide 5.0. The binding site was defined by a cubic box of 10 Å in the x, y, and z directions, centered on the reference ligand. The option, allowing only for the docking of ligands containing a defined range of atoms, was disabled; thus, all compounds were docked independently from the number of their atoms; whereas the GLIDE [39] defaults were used for all of the other settings. For each ligand, two different docking analyses were performed by using the standard precision (SP) and the extra precision (XP) methods. The XP mode is a refinement tool that is designated to be employed only on good ligand poses; the sampling is based on an anchor and refined growth method, and the scoring function consists of a more complete treatment of some of the SP terms, such as the hydrophobic and solvation terms.

Gold 5.1. The docking site was determined as the region comprising all residues within 10 Å from the ligand in the X-ray crystal structures. The possibility for the ligand to flip ring corners was activated, while the “allow early termination” command was deactivated. The GOLD [40] defaults were employed for all other parameters, and the ligands were subjected to 30 genetic algorithm runs. Four different docking analyses were performed. The four fitness functions implemented in the GOLD package, i.e. Astex Statistical Potential (ASP), ChemScore (CS), GoldScore (GS), and ChemPLP (PLP), were used

Plants. This docking software use Ant Colony Optimization, a state-of-art global optimization algorithm to find the minima of a scoring function representing a favorable complex structure [41]. The ChemPLP scoring function was employed to score protein–ligand interactions, as well as intra-ligand clash terms. Default setting for all parameters were employed for the scoring function, as well as the optimization algorithm (search speed setting: “speed1”). The region of interest used by PLANTS [42] was determined by considering the bound ligand as the center of a grid box of 10 Å in the x, y, and z directions.

rDOCK 1.0. This docking program uses a combination of stochastic and deterministic search techniques to generate low-energy ligand poses [43]. The docking protocol generates a single-ligand pose, using three stages of Genetic Algorithm search (GA1, GA2, GA3), followed by low-temperature Monte Carlo (MC) and Simplex minimization (MIN) stages. The GA stages are independent, and they are designed to be used sequentially. The cavity within a radius of 10 Å from the reference ligand was used to represent the binding site; for all the other parameters, rDOCK defaults were employed.

Autodock Vina 1.1. All the input files (ligands and protein) originating from AUTODOCK Tools for the AUTODOCK calculations were also used for the AUTODOCK VINA [44] calculations, including the grid box dimension. The exhaustiveness parameter was set to 10, and the Energy_range was set to 1, whereas all other parameters were used as their default. The scoring function implemented in VINA combines some advantages of knowledge-based potentials and empirical scoring functions, extracting information from both the conformational preferences of the receptor–ligand complexes and the experimental affinity measurements.

### 3.4. Docking Score Evaluation

By applying the 13 docking procedures described above, each ligand was docked into the 60 protein binding sites. Initially, the docking results obtained by different docking procedures were analyzed separately. For each docking calculation (i.e., each ligand docked into each target), the best-scored pose was considered as the docking result. The corresponding docking scores were collected; then, for each target, docking scores of the 600 docked ligands were normalized, based on the minimum and maximum score values obtained (i.e., the minimum and maximum docking scores estimated among the docking results of all 600 ligands within the particular target). Subsequently, the aforementioned normalized score values of the 60 targets queried by a specific ligand were compared with one another, in order to estimate the relative affinities of the 60 targets for the specific ligand, and therefore to determine the rank position of the correct target. The ranking position of the decoy targets for the specific ligand were thus estimated accordingly. By considering the individual ranking position reached by the correct target for each of the 600 ligands, we calculated the median ranking position of the targets achieved by each different docking procedure. Likewise, the standard deviation (SD) of the obtained results was calculated. The median ranking position and SD were taken into consideration to compare the performances of each docking procedure (see Results and Discussion for details).

### 3.5. Consensus Docking Analysis

To perform a consensus docking analysis, results obtained through different docking procedures were compared between one another, using a previously developed protocol [29]. We then estimated the number of docking procedures able to classify the proper target of each ligand in the top-ranked 10% of the total targets (i.e., the consensus level). In other words, we considered as positive only those methods that ranked the correct target of a ligand in the first six positions out of the total 60. The same procedure was applied to the 59 unrelated target of ligands, and likewise, the consensus level was calculated for each of them. Once again, the 60 consensus level values calculated for each ligand (for both the proper and unrelated targets) were ranked, and the ranking position of each proper target was estimated. The median of the 600 individual ranking positions of the proper target of each ligand was calculated. This value estimated the target prediction ability of the consensus docking analysis (see Results and Discussion for details). Moreover, the total consensus level of each target was calculated by considering the median consensus level values obtained for the 10 ligands of the same target (see Table 3, Results and Discussion).

### 3.6. Evaluation of True Positive Rate and False Discovery Rate

The TPR of target-fishing performance has been calculated for each of the 13 docking methods tested, as well as for the consensus docking approach, using the following equation:

(1) $$\frac{nTP}{nTD}=\frac{nTP}{600}$$

where *nTP* is the number of true positives (i.e., the event that the true target of a ligand is ranked in top 10% of the targets dataset), and *nTD* is the number of true dockings (the number of correct ligand-protein combinations, corresponding to 600). The false discovery rate (FDR) of the target-fishing performance has been calculated for each of the 13 docking methods tested, as well as for the consensus docking approach, using the following equation:

(2) $$\frac{nFP}{nPP}=\frac{nFP}{3600}$$

where *nFP* is the number of false positives (i.e., the event that the non-true target is ranked in top 10% of the targets dataset) and *nPP* is the number of predicted positives (i.e., the total number of targets predicted in the top 10% of the targets dataset considering all 600 ligands, corresponding to 3600). Both the TPR and FDR values were reported as percentages of the maximum achievable values.

## 4. Conclusions

In this study, the reliability of a docking-based TF approach was evaluated through an extensive docking study. A benchmark dataset of 60 targets and 600 known-active ligands was generated and used to assess the ability of 13 docking procedures for identifying the proper target of each ligand. The distinct analyses of the different docking methods showed a performance rating corresponding to an overall a success rate of around 25–35%, not overcoming 36% of true predictions. A performance comparable to that shown by the best tested methods was observed by applying a consensus docking strategy combining the results of multiple docking procedures. Although the approach did not result in a significant improvement of protein target prediction capabilities, and it was not able to reduce the variability of results obtained across the range of different target proteins, consensus docking highlighted that the results of the target prediction were deeply related to the volume and shape of the target binding site. Actually, our consensus docking-based TF protocol proved to be effective in identifying the true target of the ligands, whose corresponding receptors were characterized by a small and mainly closed binding site. To the best of our knowledge, this study represents the first extensive performance assessment of a docking-based TF approach, and the first application of consensus docking to TF strategies. The results herein reported thus allow preliminary clues to be figured out for the applicability domain of this receptor-based strategy, where the variability of the results should be reduced, thanks to the higher reliability of the target predictions. Indeed, consensus docking-based TF could be profitably applied, with the aim of evaluating the possible affinity of a ligand of interest for a dataset of potential target receptors presenting small or enclosed binding cavities. In this case, the protocol should only generate valuable predictions, and the proteins ranked in the top-scored positions of the dataset used could be reliably considered to be potential targets of the query ligand. Otherwise, the protocol could be even applied by using a comprehensive database of the target receptors, such as a whole set of X-ray structures gathered in the Protein Data Bank, regardless of the specific features of the protein binding sites. However, in this case, it should be considered that the reliability of the predictions would depend on the shape and volume of the protein-binding pockets taken into account. In particular, the probability that a protein that is suggested as a potential target of the query ligand is an actual target receptor of that ligand would be inversely proportional to the volume and solvent accessibility of that specific protein-binding site. We are conscious that further evaluations on the effectiveness of this approach are still necessary, to better elucidate its strengths and limitations, as well as to understand how to improve the reliability of this procedure, thus expanding its range of applicability. Nevertheless, the study herein reported paves the way for the development of efficient TF strategies, based on docking methods and their combined applications.

## Figures and Tables

**Table 1:** A list of the 60 targets selected from the DUD, MUV, and ChEMBL datasets. The typology and PDB code [18] of each target are listed in the table.

Type	PDB Code	Source
Acetylcholinesterase (ACHE)	1EVE	DUD
Aldose Reductase (ALR2)	1AH3	
AmpC beta-lactamase (AmpC)	1XGJ	
Androgen Receptor (AR)	1XQ2	
Cyclic dependent Kinase 2 (CDK2)	1CKP	
Cyclooxigenase 1 (COX1)	1P4G	
Cyclooxigenase 2 (COX2)	1CX2	
Dihidrofolate Reductase (DHFR)	2DFR	
Epidermal Growth Factor Receptor (EGFr)	1M17	
Estrogen Receptor Alpha (ERagonist)	1L2I	
Estrogen Receptor Alpha (ERantagonist)	3ERT	
Fibroblast Growth Factor Receptor 1 (FGFr1)	1AGW	
Coagulation Factor XA (FXa)	1F0R	
GAR Transformylase (GART)	1C2T	
Glycogen Phosphorylase (GPB)	1AI8	
Glucocorticoid Receptor (GR)	1M2Z	
HIV-1 protease (HIVPR)	1HPX	
HIV-1 reverse transcriptase (HIVRT)	1RT1	
HMG-CoA reductase (HMGR)	1HW8	
Heat shock protein 90 (HSP90)	1UY6	
Enoyl reductase (InhA)	1P44	
Mineralocorticoid Receptor (MR)	2AA2	
Neuraminidase (NA)	1A4G	
p38 MAP Kinase (P38 MAP)	1KV2	
poly(ADP-ribose) Polymerase (PARP)	1EFY	
Purine Nucleoside Phosphorylase (PNP)	1B8O	
Peroxisome proliferator-activated receptor gamma (PPARg)	1FM9	
Progesterone receptor (PR)	1SR7	
Retinoic acid receptor RXR-alpha (RXRa)	1MVC	
S-adenosylhomocysteine Hydrolase (SAHH)	1A7A	
Tyrosine-protein kinase Src (SRC)	2SRC	
Thrombin	1BA8	
Thymidine Kinase (TK)	1KIM	
Beta-Trypsin	1BJU	
Vascular Endothelial Growth Factor Receptor 2 (VEGFr2)	1VR2	
Cathepsin G	1KYN	MUV
Coagulation Factor XI (FXI)	4D76	
Focal Adhesion Kinase (FAK)	4Q9S	
Muscarinic Acetylcholine Receptor M1	5CXV	
cAMP-dependent Protein Kinase (PRKACA)	5BX6	
Sphingosine 1-Phosphate Receptor 1 (S1PR1)	3V2W	
Aurora kinase A	4ZS0	ChEMBL
Beta-2 Adrenergic Receptor	3P0G	
Beta-secretase 1	4RCD	
Cathepsin B	3AI8	
Cathepsin L	5F02	
Cathepsin S	4PE6	
Dopamine Receptor D3	3PBL	
Glycogen Phosphorylase	3DD1	
Insulin-like Growth Factor 1 Receptor (IGF1R)	5HZN	
Leukotriene A-4 Hydrolase	5BPP	
Macrophage Colony-Stimulating Factor 1 Receptor	4RTH	
Muscarinic Acetylcholine Receptor M3	4DAJ	
OX2 orexin receptor	4S0V	
Receptor-type tyrosine-protein kinase FLT3	4RT7	
Renin	4RYC	
Serine/threonine-protein kinase B-Raf	5JRQ	
Thymidylate synthase	5IOQ	
Tyrosine-protein Kinase ABL1	4ZOG	
Vasopressin Receptor V1R	1YTV	

**Table 2:** True positive rate (TPR) and false discovery rate (FDR) values obtained for the 13 tested docking procedures, and for the consensus docking approach. Both the TPR and FDR values are reported as percentages of the maximum achievable values.

Docking Procedures	TPR (%)	FDR (%)
Autodock	27%	76%
Fred	36%	69%
Dock6	25%	79%
Glamdock	31%	73%
GlideSP	36%	67%
GlideXP	30%	73%
GoldASP	28%	73%
GoldCSCORE	29%	74%
GoldGSCORE	25%	77%
GoldPLP	31%	72%
Plants	33%	70%
rDock	28%	76%
Vina	22%	81%
Consensus Docking	36%	67%

**Table 3:** The consensus docking (score) results of the 60 targets. The consensus level represents the number of docking procedures that are able to rank the proper target of each ligand into the top 10% of the considered targets. For each target, the consensus level is the median of the single consensus values calculated for its 10 corresponding ligands.

Target	Consensus Level	Target	Consensus Level
ACHE	2	SRC	2
ALR2	1.5	THROMBIN	6
AMPC	2	TK	11
AR	12	TRYPSIN	10
CDK2	2	VEGFR2	1.5
COX1	9	ABL1	2.5
COX2	6	B-Raf	4.5
DHFR	4	Cathepsin B	1
EGFR	1.5	Cathepsin L	2
ER_ago	12	Renin	6.5
ER_ant	10.5	Glycogen Phosphorylase	2
FGFR1	1	Cathepsin S	1
FXA	7.5	Beta-secretase 1	3
GART	11.5	FLT3	5
GPB	4	McsFR-1	1.5
GR	11	OX2	4
HIVPR	7	D3	1
HIVRT	9	V1R	1.5
HMGA	5	Cathepsin G	1.5
HSP90	2.5	Aurora kinase A	2
INHA	0	M3	1.5
MR	12	IGF1R	2
NA	11.5	Leukotriene A-4 Hydrolase	5.5
P38 MAP	2.5	Thymidylate synthase	6.5
PARP	3	S1PR1	3
PNP	3	β2 Receptor	1.5
PPARG	6	FXI	0
PR	11	FAK	1
RXR	12	PRKACA	2.5
SAHH	10	M1	1.5

## Supplementary Materials

Supplementary materials can be found at https://www.mdpi.com/1422-0067/20/5/1023/s1.
