# Supplementary material for: Extensive Reliability Evaluation of Docking-Based Target-Fishing Strategies
Source: Int J Mol Sci. 2019 Feb 27;20(5):1023. doi: 10.3390/ijms20051023 (PMC6429110; doi:10.3390/ijms20051023)
Supplement: Supplementary file 1 [file ijms-20-01023-s001.pdf]

Extensive reliability evaluation of docking-based target-fishing strategies

Margherita Lapillo,,† Tiziano Tuccinardi,† Adriano Martinelli,† Marco Macchia,† Antonio Giordano,ϕ,§ Giulio Poli*,†*

† Department of Pharmacy, University of Pisa, 56126 Pisa, Italy.

ϕ Sbarro Institute for Cancer Research and Molecular Medicine, Center for Biotechnology, College of Science and Technology, Temple University, Philadelphia, 19122, PA, USA.

§ Department of Medical Biotechnologies, University of Siena, 53100 Siena, Italy.

Address for correspondence: Giulio Poli, E-mail: giulio.poli@unipi.it and Margherita Lapillo, E-mail: margherita.lapillo@farm.unipi.it

Table of Contents

Figure S1. Consensus docking *vs.* ligand MW and fraction of sp3 carbons — Page S2

Figure S2. Consensus docking *vs.* ligand heavy atoms and aromatic heavy atoms — Page S2

Figure S3. Consensus docking *vs.* ligand charge and logP — Page S3

Figure S4. Consensus docking *vs.* ligand H-bond acceptors and donors — Page S3

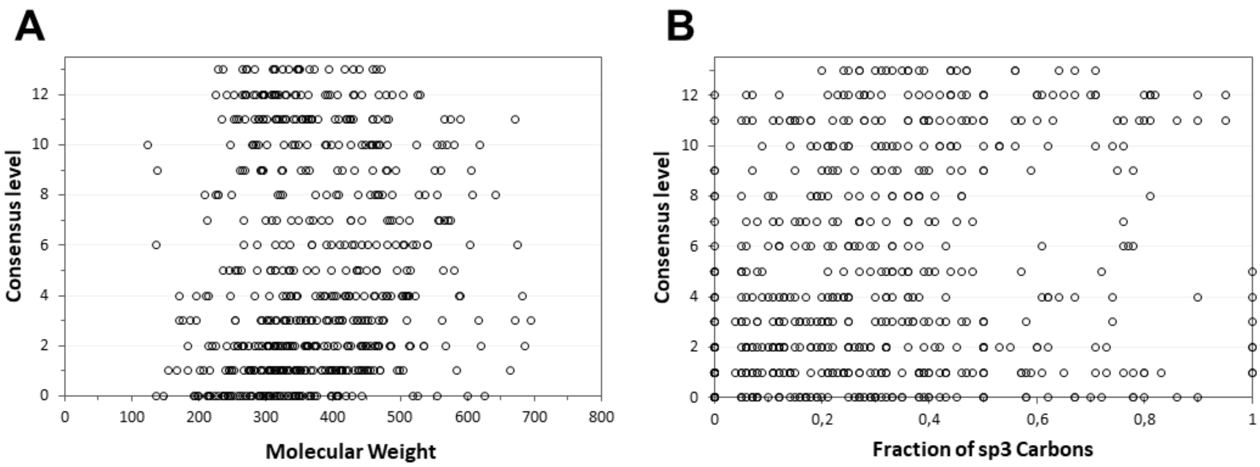

Figure S1. Analysis of consensus docking results in relation to ligand properties. Full data plots relative to (A) molecular weight and (B) fraction of sp3 carbons are reported, respectively.

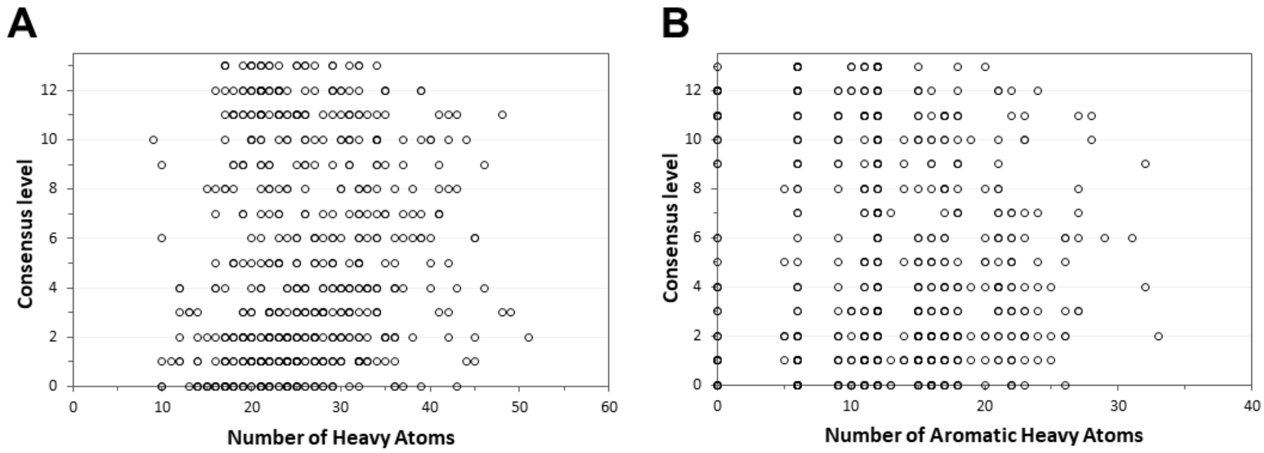

Figure S2. Analysis of consensus docking results in relation to ligand properties. Full data plots concerning (A) number of heavy atoms and (B) aromatic heavy atoms are reported, respectively.

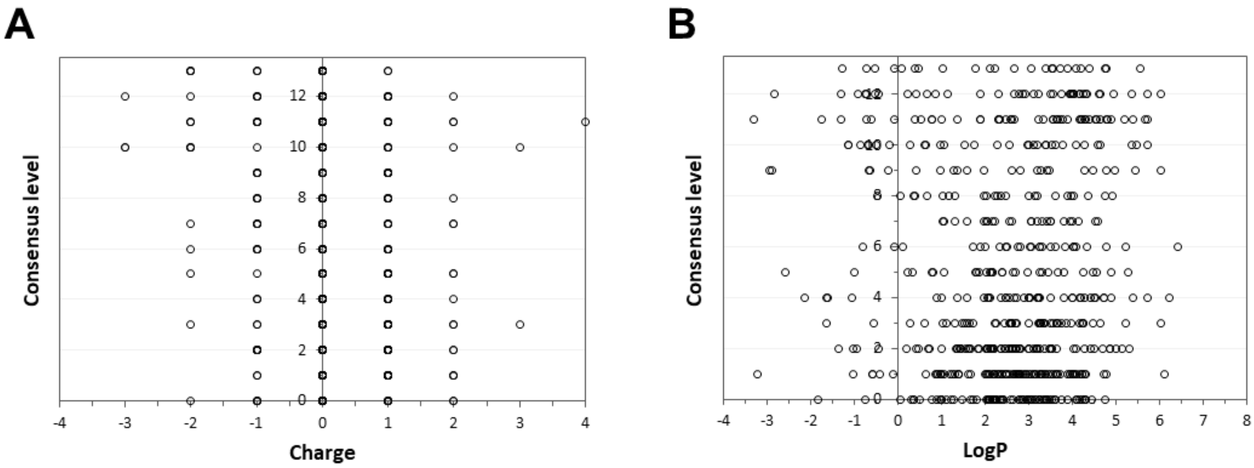

Figure S3. Analysis of consensus docking results in relation to ligand properties. Full data plots relative to (A) Charge and (B) LogP are reported, respectively.

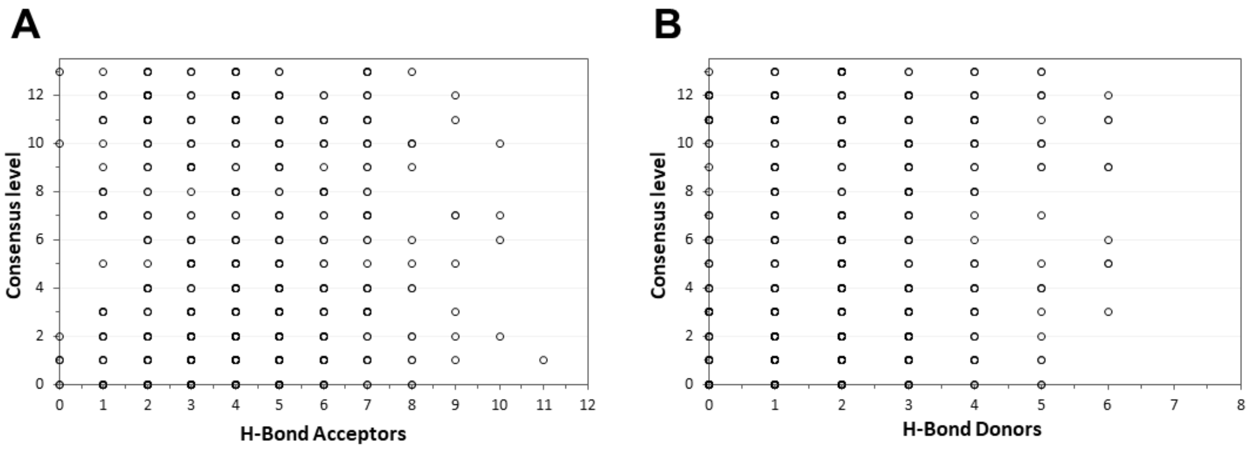

Figure S4. Analysis of consensus docking results in relation to ligand properties. Full data plots concerning (A) number of H-bond acceptors and (B) number of H-bond donors are reported, respectively.
